# Testing grain size dependent variability of cosmogenic nuclide concentrations for isochron burial dating of fluvial sediment (Pannonian Basin, Hungary)

**DOI:** 10.1016/j.mex.2025.103708

**Published:** 2025-11-07

**Authors:** Zsófia Ruszkiczay-Rüdiger, Gábor Csillag, Alexander Wieser, Oscar Marchhart, Mihály Braun, László Fodor

**Affiliations:** a HUN-REN Research Centre for Astronomy and Earth Sciences, Institute for Geological and Geochemical Research, 1112 Budapest, Budaörsi út 45, Hungary; b CSFK, MTA Centre of Excellence, Konkoly Thege Miklós út 15-17, 1121 Budapest, Hungary; c University of Vienna, Faculty of Physics, Isotope Physics, Währinger Straße 17, 1090 Vienna, Austria; d HUN-REN Institute of Nuclear Research (Atomki), 4026 Debrecen, Bem tér 18/c, Hungary; e HUN-REN Institute of Earth Physics and Space Science, 9400 Sopron Csatkai E. u. 6-8, Hungary; f Eötvös Loránd University (ELTE), Institute of Geography and Earth Sciences, Department of Geology, Budapest 1117 Pázmány P. sétány 1/C, Hungary, Hungary,

**Keywords:** Isochron burial dating, Cosmogenic radionuclides, Aluminium-26, Beryllium-10, Clast size dependent concentrations, Age determination, Fluvial sandy gravel

## Abstract

In this study a new approach of cosmogenic radionuclide (CRN) isochron burial dating was developed for locations where the maximum clast size of the sediment does not reach the size of using single cobble samples. This modification makes use of the presumption that different grain size fractions have diverse pre-burial histories and thus are deposited with diverse amount of CRNs. Accordingly, a bulk sample is taken at a certain sediment depth, from which several grain size fractions are selected. These are treated as individual samples for isochron burial age determination. To test our hypothesis, a fluvial sediment succession of the Paleo-Danube River in the Western Pannonian Basin, with an assumed Pliocene age was sampled at two sample depths. The different grain size fractions (0.25-0.5; 1-2; 4-8; 10-20; and 40-60 mm; n=7) provided variable ^26^Al and ^10^Be concentrations, supporting the validity of our assumption. The difference between the lowest and largest concentrations was limited making age determination and outlier identification more challenging compared to an isochron with more variable CRN concentrations. After a careful outlier identification, χ^2^ minimisation inverse modelling yielded 3.9 ± 0.7 Ma as the age of fluvial sedimentation.

The offered sampling strategy opens a window towards further application of the CRN isochron burial dating.

The burial age of fluvial sediments that were hitherto undatable due to their limited thickness and small clast size can be determined.

After the presented adjustment of the sampling strategy the age of deposition can be calculated by the standard ways of isochron burial dating.

## Graphical abstract

**Figure:**
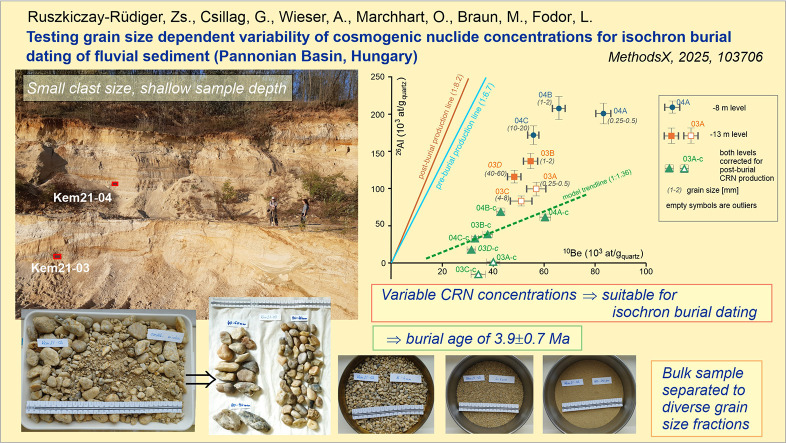

Specifications table

**Table:** 

Subject area	Earth and Planetary Sciences
**More specific subject area**	Geochronology
**Name of your method**	Isochron burial dating using cosmogenic radionuclides
**Name and reference of original method**	[1]. An isochron method for cosmogenic nuclide dating of buried soils and sediments, Am. J. Sci., 308, 1083–1114, https://doi.org/10.2475/10.2008.02. [2]. Rock uplift rates in South Africa from isochron burial dating of fluvial and marine terraces, Geology, 40, 1019–1022, https://doi.org/10.1130/G33172.1. [3]. Dating the Homo erectus bearing travertine from Kocaba¸s, (Denizli, Turkey) at least 1.1 Ma, Earth Planet. *Sc*. Lett. 390, 8–18, https://doi.org/10.1016/j.epsl.2013.12.031. [4]. Early Pleistocene presence of Acheulian hominins in South India, Science, 331, 1596–1599, https://doi.org/10.1126/science.1200183.
**Resource availability**	Not applicable.

## Background

**Fig. 1:**
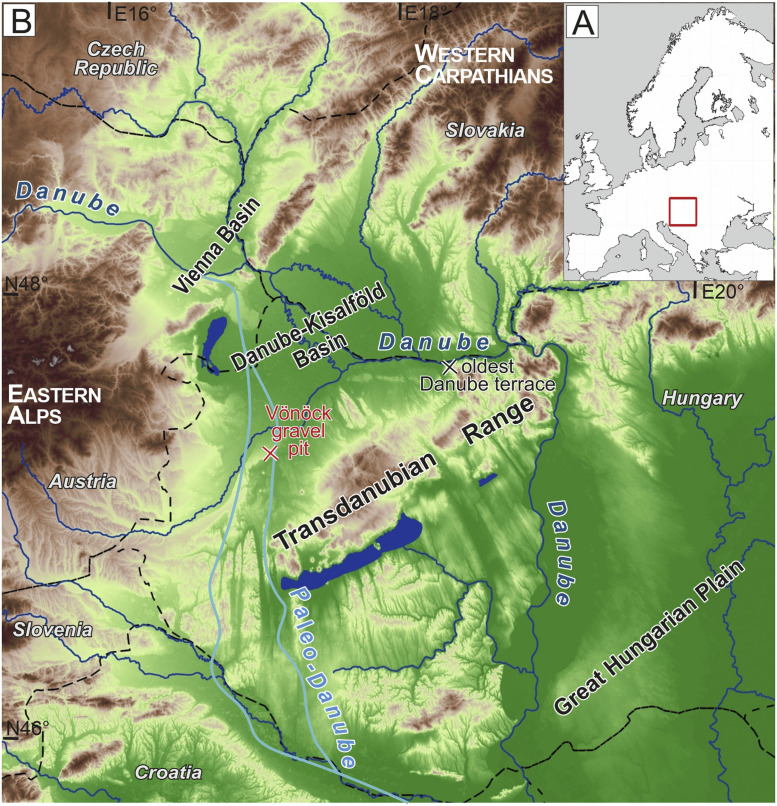
A: Location of the Pannonian Basin in Europe. B: The western Pannonian Basin with the major rivers and the course of the Paleo-Danube River. Red cross indicates the study site, black cross is the location of the oldest terrace of the Danube along its modern course (∼2.9 Ma [5],).

The CRN isochron burial dating method was designed for the age determination of sediments, where the shallow burial depth does not allow complete shielding from cosmic irradiation [1,2]. It conventionally consists of taking several cobbles from the same stratigraphic level at the same subsurface depth, which are large enough to be treated as individual samples. The isochron approach is based on the presumption that the cobbles arrive from diverse source areas with different inherited CRN inventories and after deposition they share the same post-burial history. However, the maximum clast size of the quartz-rich cross-bedded gravel and sand exposed in the gravel pit near Vönöck village (Lat: 47.32822°, Long: 17.166722°, 160 m asl; Fig. 1) did not allow this approach. Our methodological development focussed on customization of the method on fluvial sediments lacking large enough cobbles to be treated as individual samples for the conventional isochron burial dating.

The Vönöck location is in the southern part of the Danube-Kisalföld Basin, in a key position for the age determination of the timing of the last milestone of the landscape evolution of the Western Pannonian Basin. The outcropping sediment was deposited by the Paleo-Danube River crossing the area in a north-south direction after the fill-up of the Lake Pannon. Magnetic polarity and biostratigraphy combined with correlations using seismic reflection profiles of borehole data set the maximum age of the sediment at ∼7 Ma [[6], [7], [8]]. The alluvial sedimentation ceased by the last major drainage pattern reorganization causing the abandonment of the north-south course (Fig. 1). The minimum age of the shift of the Paleo-Danube towards its present course has been settled by the oldest Danube terrace along its modern, west-east trending valley, dated to 2.9 ± 0.5 Ma [5]. The alluvial sediment at Vönöck is supposed to be the last member of the fluvial sediments deposited by the southward flowing Paleo-Danube. Therefore, its age determination can provide a better constrain of the maximum age of the drainage reorganization, which information is crucial for the palaeosurface reconstruction and landscape evolution modelling [9].

Several studies reported variable CRN concentrations for different grain sizes of sand and pebbles in river sediments due to diverse provenance and denudation history [[10], [11], [12], [13], [14]]. This difference in the amount of inherited CRN inventories at the time of sediment deposition may serve as a solid basis to use different grain sizes as individual samples for isochron burial dating. Accordingly, our sampling approach consisted of taking bulk samples from two sampling depths from which different grain sizes were selected and treated as individual samples (Table S1). If the differences of the inherited CRN concentrations between the different grain sizes are large enough, the ^10^Be and ^26^Al concentrations of these samples can be used to calculate the isochron burial age of the sediment.

## Method details

The presumed Pliocene time range of sediment deposition at Vönöck is well suited for the burial age determination method using the CRN pair of ^26^Al and ^10^Be. These radionuclides have different half-lives and are produced in quartz exposed to secondary cosmic rays by different production rates but with a fixed ratio at the surface, but variable with depth due to the muogenic production taking a dominant role [15,16] (Table 1). The cosmic rays are rapidly attenuated with depth, therefore if a formerly exposed quartz grain is eroded and buried, the amount and ratio of CRNs start to decrease, providing the basis for the burial age determination of the sediment up to ∼5 Ma [17,16]. However, in case of shallow burial (less than 15–20 m depth), typical for fluvial sediments, the CRN production does not stop but is continued at a decreased rate. In such cases post-burial production must be accounted for. The isochron burial dating method was developed to deal with post-burial production. It normally uses a set of individual clasts as discrete samples from the same sediment horizon that share the same post-burial history but arrived with different CRN concentrations [1,2]. Here we use isochron burial dating by χ^2^ fitting inverse modelling [3,4,18]. This method considers the subsurface depth of the samples, and together with the burial age, it allows the determination of the source and sink denudation rates. As the samples are coming from two different depth levels the applied method combines the above-described advantages of isochron dating with those of the depth profile dating based on considering the decreasing CRN production rates at increasing sample depth [[5], [19], [20], [21], [22]]. Both techniques use the same set of equations and can be simultaneously solved by χ^2^ fitting inverse modelling for all samples, as it was described by Ruszkiczay-Rüdiger et al. [18]. It must be noted that the source denudation rates can only be regarded as apparent, because this parameter accounts for the CRN concentrations at the time of deposition, which is modulated not only by the surface denudation at the source, but also by the CRN production rates (depending on elevation and latitude of the catchment). Variables and constants used for the burial age determination in this study are found in Table 1.

**Table 1:** Variables and constants used for isochron burial age inverse modelling (qtz: quartz; SLHL: sea level high latitude).

Model parameter [unit]	Value	Reference
^10^Be spallogenic production rate SLHL [atoms/g_qtz_/yr]	4.01 ± 0.33	Borchers et al. [23]
Scaling of muogenic production rates	Heisinger et al. [[24], [25]] modified by Balco [15], calculated using the code of Nørgaard et al. [26]	
Attenuation lengths (neutrons, thermal muons, fast muons) [g/cm^2^]	160, 1500, 4320	Heisinger et al. [[24], [25]]; Braucher et al. [27]
Scaling of spallogenic production rates	time independent	Lal [28]/Stone [29]; calculated using Vermeesch [30]
^10^Be half-life [yr]	1 387 000 ± 12 000	Chmeleff et al. [31]; Korschinek et al. (2010);
^26^Al half-life [yr]	705 000 ± 17 000	[32]; Nishiizumi [33]
production rate ratio ^26^Al/^10^Be	6.7 ± 0.6	Fenton et al. [34]
Bedrock density at source [g/cm³]	2.5	estimated value
Gravel density [g/cm³]	2 ± 0.2	estimated value
Source coordinates	Lat N: 48°, Long E: 14.5°, 700 m a.s.l.	estimated for the catchment area of the Danube
Upper threshold of sink denudation rate [m/Myr]	50	Based on Ruszkiczay-Rüdiger et al. [21,5]

For the description of the isochron burial age calculations using the χ^2^ fitting inverse modelling and the equations used for age determination refer to Ruszkiczay-Rüdiger et al. [18]. A user-defined upper threshold value was set to the sink denudation rate at 50 m/Ma, a value 2.5 times larger than the highest denudation rates calculated using ^10^Be depth profiles (∼20 m/Ma) on Danube terraces in the north-western part of the Pannonian Basin with similar lithology, topography and climate conditions [[5], [21]].

**Fig. 2:**
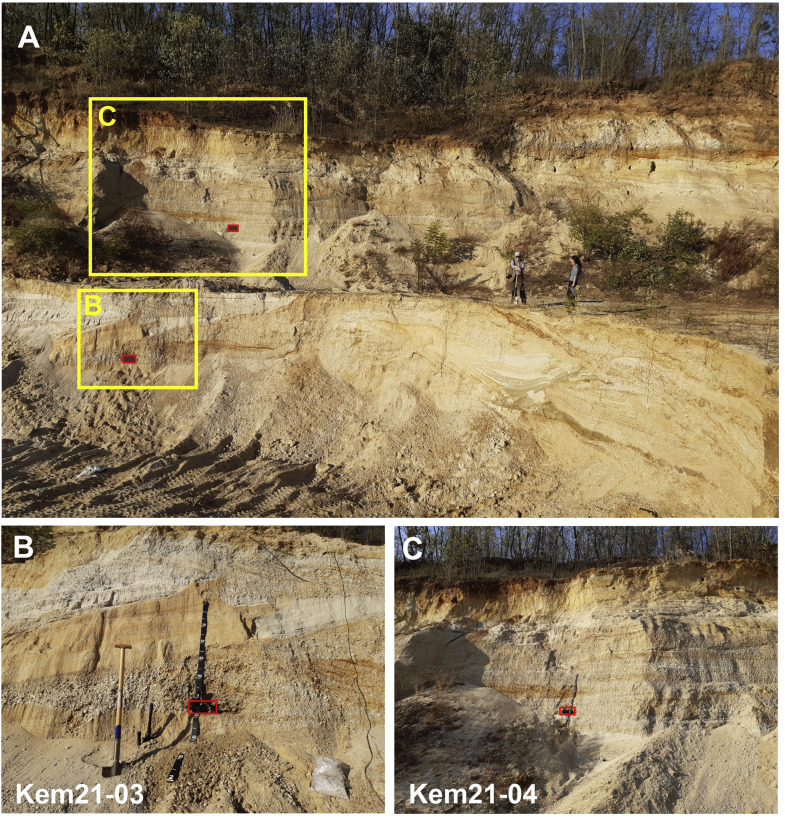
A) Overview of the gravel pit at Vönöck with the sample locations (red rectangles). B) The Kem21–03 sample at ∼13 m subsurface depth. C) The Kem21–04 sample at ∼8 m subsurface depth.

The sediment was sampled in 2021 at two quarry levels: a lower layer close to the bottom of the quarry at ∼13 ± 2 m (Kem21–03) and an upper layer at 8 ± 2 m depth (Kem21–04) with a maximum grain size of 6–8 cm and 1–2 cm, respectively. The uncertainty of the sample depth comes from the anthropogenic disturbance of the surface around the gravel pit. No hiatus or unconformity was observed between the two sampled horizons; therefore, the sediment is considered to belong to a continuous fluvial sedimentation, with no considerable difference in age between its upper and lower parts (Fig. 2). The small maximum grain size does not allow to use individual clasts as sub-samples for an isochron sample set. Following our novel approach the sediment was sampled for the extraction of different grain sizes from the same sedimentary unit. Accordingly, ∼3 kg of bulk sample was collected from both sampled levels, which was sieved into several grain size fractions, among which 4 (0.25–0.5; 1–2; 4–8; 40–60 mm) from the lower (Kem21–03) and 3 (0.25–0.5; 1–2; 10–20 mm) from the upper (Kem21–04) sample were selected and processed for analysis.

From the sand and small pebble fractions ∼0.2–0.5 kg was achieved by sieving, which provided a large enough number of particles for a representative sample to exclude the biassing effect of any individual clasts (at least 30 particles [35],). From the largest, 4–6 cm particle size we could sieve from the bulk sample a selection of ∼0.5 kg sample composed of ∼15 quartz and quartzite pebbles. Based on our previous experience with pebble samples used for depth profile dating [[5], [21]] we considered that the available material can be sufficient to have a representative amalgamated sample (one sample is 6–7 % of the total sample weight).

Laboratory procedures occurred in the Sample preparation laboratory of the HUN-REN CSFK, Institute for Geological and Geochemical Research, Budapest following the protocol described by Ruszkiczay-Rüdiger et al. [36] and are presented in the Supplement section 1. Accelerator mass spectrometry measurement of the isotopic ratios was carried out at the Vienna Environmental Research Accelerator at the University of Vienna (VERA; [37,38]) and is described in the Supplement section 2. Laboratory data and measurement results are presented in Tables 2 and S1. Fig. 2.

**Table 2:** ^10^Be and ^26^Al concentrations and ^26^Al/^10^Be ratio of the samples. For laboratory data and measurement results refer to Table S1.

Sample	particle size	^10^Be	1σ ^10^Be	^26^Al	1σ ^26^Al	^26^Al/^10^Be	1σ
	(mm)	(kat/g_quartz_)	(kat/g_quartz_)		^26^Al/^10^Be		
*13m depth level*							
Kem21–03A	0.25–0.5	56.9	3.8	99.5	9.1	1.75	0.20
Kem21–03B	1–2	54.7	2.8	136.8	10.4	2.50	0.23
Kem21–03C	4–8	51.0	4.3	83.2	6.7	1.63	0.19
Kem21–03D	40–60	48.3	2.6	115.9	8.5	2.40	0.22
*8m depth level*							
Kem21–04A	0.25–0.5	83.3	3.0	200.7	13.7	2.41	0.19
Kem21–04B	1–2	65.7	2.5	207.9	15.6	3.16	0.27
Kem21–04C	10–20	55.7	2.2	171.9	12.6	3.09	0.26

## Method validation

In order to properly judge the suitability of the alternative sampling technique for the isochron burial dating applied by this study, at first there is a need to look at the measured CRN concentrations. If these prove to be different it potentially makes the dataset suitable for the calculation of an isochron burial age. As a next step, the dataset has to pass a careful scrutiny for the identification of potential outliers [18]. The remaining set of samples can be used for the calculation of the most probable burial age. Aiming at a proof of the aptness of our alternative sampling strategy, in the following the above outlined steps are presented on the example of the Vönöck sample set.

**Fig. 3:**
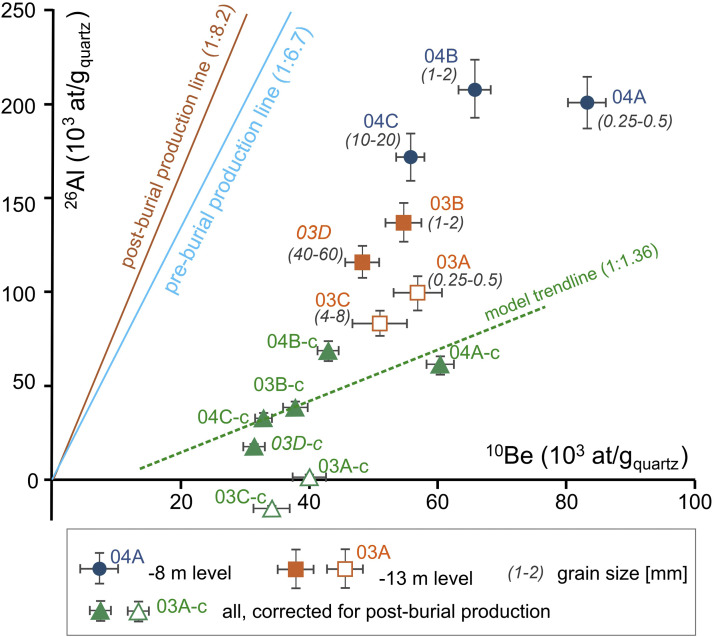
^10^Be concentrations plotted against the ^26^Al concentrations of the samples at the two sampled quarry levels (Tables 2, S1). Note that sample codes appear without the “Kem21-“ prefix for simplicity. Empty signs are the outliers. The pre-burial production line is at the ∼6.7: 1 spallogenic ^26^Al/^10^Be production rate ratio at the surface. The post-burial ^26^Al/^10^Be line represents the production rate ratio at the measured sample depth (∼8.2: 1, the mean of the ∼8.4: 1 and ∼8.0: 1^26^Al/^10^Be ratios, relevant for the subsurface depth of the upper and lower sampled levels). Green triangles are samples with the measured CRN concentrations corrected for the post-burial inventories, rendering them suitable to fit a trendline as an isochron. Notethat the correction for the post-burial CRN production for the low ^26^Al concentration Kem21–03A and-03C samples led to zero or negative ^26^Al concentrations. Accordingly, all the measured ^26^Al inventory of these samples was produced after deposition and no pre-burial concentrations have remained due to radioactive decay. This makes them irrelevant for burial age determination.

The results of the AMS measurements, the calculated ^26^Al and ^10^Be concentrations, the ^26^Al/^10^Be ratios and simple burial ages are presented in Tables 1; S1, Fig. 3. The different grain size fractions provided variable CRN concentrations (∼48–83 kat/g_quartz_ and ∼83–208 kat/g_quartz_ for ^10^Be and ^26^Al, respectively) with somewhat higher values in the upper level compared to the lower level, in agreement with the larger post-burial production at shallower depth. As all clast sizes of a bulk sample shared the same post-burial history, the measured difference must stem from their history prior to deposition and burial. This supports our presumption based on previous studies, that distinct grain size fractions have diverse source areas and thus arrive with variable CRN concentrations (e.g. [11,12]), making our dataset suitable for isochron burial dating.

The ^26^Al/^10^Be ratios vary between 1.6 ± 0.2 and 3.2 ± 0.3. The CRN concentrations show a decreasing trend towards the larger grain sizes at each level. This tendency is only broken for ^26^Al by the Kem21–03A and −03C samples. These are the samples with the lowest ^26^Al/^10^Be ratios (Fig. 3). The scatter of the ^26^Al/^10^Be ratios calls the attention on the possible presence of outliers in the dataset. Nevertheless, the difference between the lowest and largest concentrations was limited: 42 % for ^10^Be and 60 % for ^26^Al, which might make age determination and outlier identification more challenging compared to an isochron with a wider spread of CRN concentrations.

The statistical integrity of the samples was examined using the reduced χ^2^ test [39]. This indicated 3 samples, those with the highest and lowest ^26^Al/^10^Be ratio (Kem21–03A, −03C and −04B) as potential outliers. However, the ^26^Al/^10^Be ratio is only one component of the isochron burial age determination and does not account for the different nuclide concentrations and different post-burial CRN inventories at the two sampled depth levels. Aiming at a robust age determination, two more tests were applied on the dataset: (1) the isochron burial age was estimated iteratively via bootstrapping to identify samples that considerably bias the burial age; (2) the post-burial component of the CRN concentrations was determined for both levels for all burial age scenarios to check how the ratio of the pre- and post-burial inventories of the measured CRN concentrations (Table S2; for methodology refer to Ruszkiczay-Rüdiger et al., 2005).

**Fig. 4:**
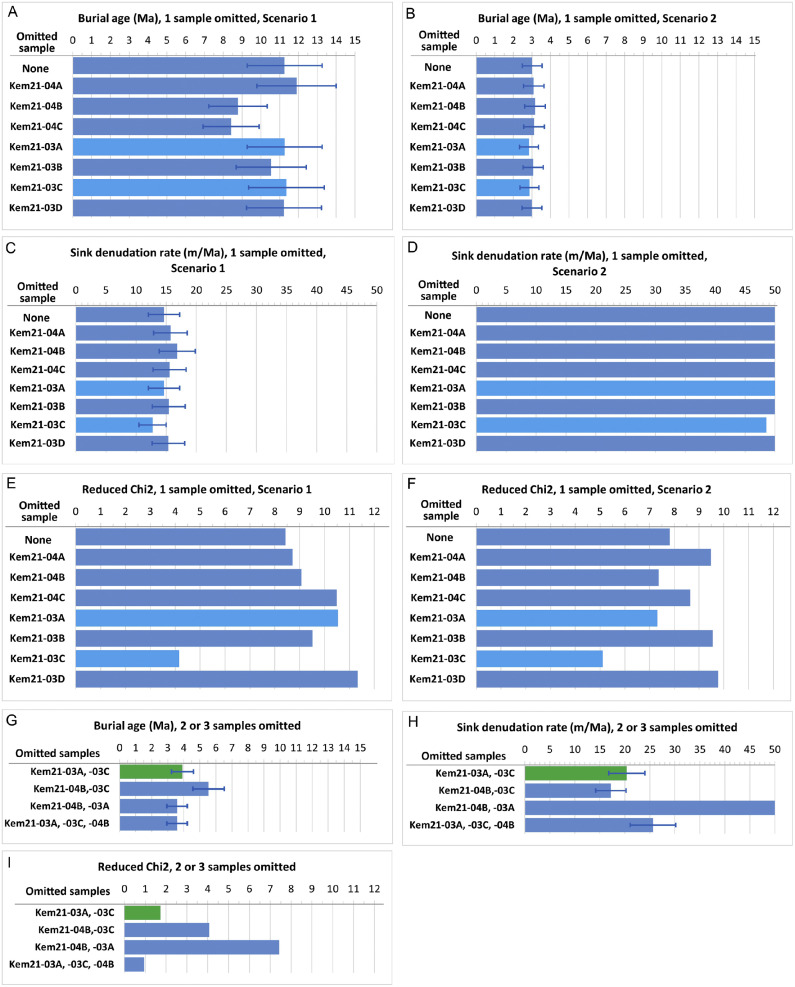
Histograms summarising the results of the bootstrap test (Table S2). When all samples were included or a single sample was excluded two sets of burial age (A, B) – sink denudation rate (C, D) pairs provided similar goodness of fit (F, F), therefore these are presented as Scenario 1 (A, C, E) and Scenario 2 (B,D,F). The runs with the omission of the samples previously flagged as potential outliers are marked with lighter blue colour (Kem21–03A and −03C). The model fit improved significantly with the omission of both these samples. A few runs with the exclusion of a third potential outlier sample are also presented (G, H, I). The two cases with the best model fit (smallest Rχ^2^ on inset I) provided burial age-denudation pairs in agreement within uncertainties (G, H), among which the one with the omission of two samples only was accepted as the most probable solution (green colour on G, H, I). Note that for bootstrap runs where sink denudation rate reached the upper threshold value of 50 m/Ma, the uncertainty estimates are irrelevant, therefore are not printed on the histograms.
(ii) Scenario 2: Burial ages at or under the minimum age constraint of the model (∼3 Ma [5],) accompanied by sink denudation rates hitting the 50 m/Ma upper threshold of the model (Fig. 4B,D,F; Table S2). In this scenario the post-burial ^10^Be and ^26^Al inventories were reasonable, with ∼14–22 % and 40–54 % of the measured CRN concentrations, respectively.

The bootstrap test showed that in case no, or only a single sample was omitted from the dataset, it was not possible to infer a reliable and robust burial age. The resulting model solution formed two groups:

(i) Scenario 1: Burial ages above the upper age constraint (∼ 7 Ma [6],) of the sediment (modelled burial ages between ∼8 Ma and 12 Ma) coupled with source denudation rates close to zero (Fig. 4.A,C,E; Table S2). For this scenario post-burial production is ∼100 % for ^26^Al, the nuclide of faster decay and higher muogenic production rate, also making the validity of these results questionable.

In summary: the burial age denudation rate pairs when all samples are included or only one sample is omitted are geologically impossible. Besides, the model fit is poor with reduced χ^2^ (Rχ^2^) values above 8 in most cases

When two samples were omitted the model fit improved (Rχ^2^ of 4–7) (Fig. 4.G,H,I; Table S2). The omission of the Kem21–03C and Kem21–03A samples resulted in the best model fit (Rχ^2^ = 1.7), indicative of these samples as probably being out of the main trendline defined by the dataset (Fig. 3). This observation is in accordance with the statistical test showing these samples as potential outliers due to their lowest ^26^Al concentrations and ^26^Al/^10^Be ratios compared to the rest of the dataset. The burial age calculations by excluding both the Kem21–03A and −03C samples provided a plausible result: burial age is 3.91 ± 0.69 Ma coupled with a sink denudation rate of 20.4 ± 3.2 m/Ma and apparent source denudation rates between 13 and 26 m/Ma, and the model fit has also improved considerably). Provided that the no inherited ^26^Al has remained in the CRN inventory of the low ^26^Al concentration Kem21–03A and −03C samples, their exclusion as outliers from burial age determination seems to be reasonable (Fig 3, Table S2). This solution is geologically plausible, provides a good model fit and reasonable post-burial inventories. The omission of other sample pairs led to significantly worse model fit and/or still geologically unreasonable burial ages and/or sink denudation rates (Fig. 4, G, H, I; Table S2).

The scenario by excluding 3 samples was also tested with the omission of all 3 samples pinpointed by the statistical analysis (Kem21–03A, −03C, −04B, Fig. 4G, H, I). This setup led to a burial age of 3.6 ± 0.63 Ma coupled with a sink denudation rate of 25.6 ± 3.2 m/Mry and a very good model fit (Rχ^2^ = 0.9). This age overlaps with the solution provided when only the −03A and −03C samples were omitted. We consider the model solution with only two samples excluded to be more robust, therefore acceptable as the most probable burial age and sink denudation rate pair of the sediment of the Paleo-Danube at Vönöck (Table 3). The validity of the new methodology seems to be supported both by the above-described inquiry of the measurement data, and by good fit of the obtained age to the geological context.

**Table 3:** Most probable burial age of the Paleo-Danube sediments at Vönöck.

Burial age (Ma)	3.92	±	0.69
Sink denudation rate (m/Ma)	20.4	±	3.2
Source denudation rates (m/Ma)	13–16	±	2–4
R χ^2^	1.7		

To explain the very low ^26^Al concentrations of the two outlying samples the following reasons may be invoked. (i) Based on previous experiences [36] we excluded the possibility of a laboratory loss of 26Al because no anomalies in chemistry or Al Fluoride precipitates were observed during sample processing. (ii) A plausible reason for low CRN ratios may be intermittent storage during transport, that may to reduce the inherited ^26^Al/^10^Be ratio considerably [40]. (iii) The low ^26^Al/^10^Be ratio of the 0.25–0.5 grain size fraction in the bottom quarry level (Kem20–03A), however is challenging to explain, as this fraction was used from the upper quarry level as well (Kem21–04A), where it contained enough ^26^Al for dating. One reason could be that the fine and middle sand fraction in the lowest part of the sediment was mixed with the underlying Early Pliocene sandy sediments biassing the ^26^Al concentrations to such low values.

## Limitations

Main limitation of the method is the possibility that the differences of CRN concentrations between the samples taken from the different grain-size fractions of the sediment will not be large enough to properly constrain the isochron. This risk increases with both shallow burial depth and long burial time as both lead to higher post-burial inventories compared to the inherited concentrations.

Accordingly, we suggest taking the bulk samples from the deepest possible stratigraphic level of the targeted sediment, and at least from ∼5 m depth. Besides, we recommend selecting at least 5–6 grain size fractions with the widest possible spread to have the best potential for diverse pre-burial histories and thus broadly different pre-burial/inherited CRN concentrations of the sub-samples. From grain size fraction above a few cm-s, a large bulk sample may be necessary to have the minimum number of clasts in the selected size that is suitable to smooth the variability of the inherited CRN inventory of the individual clasts. We suggest that ∼15 clasts are already sufficient, but obviously selection of a larger number of pebbles may further reduce the impact of a single pebble on the mean CRN concentrations.

Lastly, taking samples at several depth levels may be useful if the sediment pile is thick, and/or the spread of the particle size fractions (or the number of large cobbles for a traditional isochron) is not wide enough. This way using χ^2^ minimization inverse modelling it is possible to exploit also the depth dependent change of CRN production and determine the age of the sediment by a combination of the depth profile and the isochron techniques (as it was also demonstrated by Ruszkiczay-Rüdiger et al. [18] for “traditional” isochron cobble sample sets).

## Ethics statements

Not applicable.

## Supplementary material *and/or* additional information [optional]

### Supplement 1. laboratory procedures

The bulk samples were sieved, and the 0.25–0.5 mm fraction was directly taken for analysis. The grain size fractions larger than 1 mm were crushed and sieved, and their 0.5–1.0 mm fraction was used. Sample processing occurred in the Cosmogenic Nuclide Sample Preparation Laboratory of the Institute for Geological and Geochemical Research (HUN-REN, CSFK; Budapest, Hungary; http://www.geochem.hu/kozmogen/Lab_en.html) following the procedures by Merchel and Herpers [41] and Merchel et al. [42] as described in Ruszkiczay-Rüdiger et al. [36]. Stable ^27^Al determination was performed by Microwave Plasma – Atom Emission Spectrometry (Agilent 4100 MP-AES) at the Institute of Nuclear Research (Debrecen, Hungary). Laboratory data are presented in Laboratory data are presented in Tables 2, S1 and Fig. 3.

### Supplement 2. cosmogenic radionuclide determination by accelerator mass spectrometry (AMS)

Purified BeO and Al_2_O_3_ were mixed with Nb powder in a ∼1:2 ratio and with Fe powder in a ∼1:1 ratio, respectively and pressed into copper cathodes. All samples were measured at the Vienna Environmental Research Accelerator (VERA) at the University of Vienna ((VERA; [37,38]). The isobar ^10^B was suppressed using a foil stack absorber, resulting in machine blank ratios of ^10^Be/^9^Be = (9.3 ± 5.5)∙10^–16^ and (1.75 ± 0.34)∙10^–15^ for this study. All samples were normalized to the SMD-Be-12 standard (^10^Be/^9^Be = (1.704 ± 0.030)∙10^–12^) [43].

For ^26^Al measurements, the ILIAMS (Ion-Laser Interaction Mass Spectrometry) setup was used, where the interfering ^26^Mg was suppressed by a green laser (2.33 eV) in a radiofrequency quadrupole on the low-energy side of the AMS system [37]. This technique enables the use of the more prolific AlO^–^ ion beam, while still achieving machine blank values of ^26^Al/^27^Al = (8.8 ± 6.3)∙10^–16^ and (1.38 ± 0.80)∙10^–15^ in this study. All samples were normalized to the SMD-Al-11 standard (^26^Al/^27^Al = (9.66 ± 0.14)∙10^–12^) [44].

The reported uncertainties include the analytical uncertainties, the uncertainty of the ^10^Be and ^26^Al half-lives, the spallogenic production rate of ^10^Be, the ^26^Al/^10^Be spallogenic production rate ratio and sediment density.

## Related research article

Csillag, G., Németh, K., Sebe, K., Telbisz, T., Ruszkiczay-Rüdiger, Z., Fodor, L., Reconstructing syn-volcanic palaeosurfaces using monogenetic volcanic landforms: a methodological study and inferences for neotectonic deformation (Western Pannonian Basin, Hungary). Submitted to Global and Planetary Change

## For a published article

None

## CRediT authorship contribution statement

**Zsófia Ruszkiczay-Rüdiger:** Methodology, Conceptualization, Investigation, Data curation, Formal analysis, Funding acquisition, Visualization, Writing – original draft, Writing – review & editing. **Gábor Csillag:** Conceptualization, Investigation. **Alexander Wieser:** Formal analysis. **Oscar Marchhart:** Formal analysis. **Mihály Braun:** Formal analysis. **László Fodor:** Conceptualization, Funding acquisition, Investigation, Supervision, Writing – review & editing.

## Declaration of competing interest

The authors declare that they have no known competing financial interests or personal relationships that could have appeared to influence the work reported in this paper.

## Data Availability

All data are presented in the manuscript and in the supplementary tables.

## Appendix Supplementary materials

Image, application 1
